# Quantum entanglement and statistics of photons on a beam splitter in the form of coupled waveguides

**DOI:** 10.1038/s41598-021-89838-5

**Published:** 2021-05-13

**Authors:** D. N. Makarov, E. S. Gusarevich, A. A. Goshev, K. A. Makarova, S. N. Kapustin, A. A. Kharlamova, Yu. V. Tsykareva

**Affiliations:** Northern (Arctic) Federal University, Arkhangelsk, Russia 163002

**Keywords:** Quantum optics, Single photons and quantum effects, Atomic and molecular interactions with photons

## Abstract

It is well known that a beam splitter (BS) can be used as a source of photon quantum entanglement. This is due to the fact that the statistics of photons changes at the output ports of the BS. Usually, quantum entanglement and photon statistics take into account the constancy of the reflection coefficient *R* or the transmission coefficient *T* of the BS, where $R + T = 1$. It has recently been shown that if BS is used in the form of coupled waveguides, the coefficients *R* and *T* will depend on the photon frequencies. In this paper, it is shown that the quantum entanglement and statistics of photons at the output ports of a BS can change significantly if a BS is used in the form of coupled waveguides, where the coefficients *R* and *T* are frequency-dependent.

## Introduction

It is well known that a beam splitter (BS) is a source of quantum entangled photons^1–4^. At the same time, quantum entanglement is the basis in new directions of quantum optics: quantum metrology^5^, quantum information^6^, etc. Quantum entanglement and changes in the statistics of photons in BS can be used in linear optical quantum computing (LOQC)^7–9^. In 2001, Knill et al. showed that using BS, phase shifters, photodetectors and single photon sources, it is possible to create a universal quantum computer (KLM protocol)^10^. Beam splitters currently can be of various types. The most common and well-known type is the prismatic BS. The prismatic BS has a drawback that is its size. Therefore, they are most often used in experiments, but not in quantum technologies. An analogue of a prismatic BS can be a BS in the form of coupled waveguides. Coupling between waveguides can be achieved when two waveguides are brought together close enough to each other so that the electromagnetic fields overlap; in this case it is a directional coupler (for example^11,12^). Coupled waveguide BS has an advantage over prismatic because it is much smaller than prismatic BS and also has many other advantages^3,9,13^.

Currently, theories describing quantum entanglement and statistics of photons at the BS output ports are based on the constancy of the main parameters of the BS: the reflection coefficient *R* and the transmission coefficient *T*, where $R + T = 1$ see e.g.^1,3–5,14–16^. Recently, the paper^17^ presented the theory of a frequency-dependent BS in the form of coupled waveguides. In this work, it was shown that if the BS is presented in the form of coupled waveguides, then the coefficients *R* and *T* depend on the frequencies of the photons entering both ports of the BS. This dependence can be significant and must be taken into account in many applications of quantum optics. For example, in the works^18,19^ it was shown that the well-known theory of Hong-Ou-Mandel (HOM) interference, based on the constancy of the coefficients $R = 1/2$ and $T = 1/2$, can be significantly changed. In addition, this change affects a fundamental understanding of the HOM effect itself. It is shown that even in the case of completely identical photons and a balanced BS in the HOM effect, the visibility $\mathcal {V}$ can differ from unity.

Thus, the problem of studying many physical characteristics of a frequency-dependent BS in quantum optics is topical. In this paper, we investigated the quantum entanglement and statistical properties of photons at the output ports of a frequency-dependent BS. It should be added that the study of the quantum entanglement of two-mode photonic states with allowance for a frequency-dependent beam splitter has not been carried out previously and is a separate problem that needs to be solved. It is shown that quantum entanglement and statistical properties of photons can be very different from the case with constant coefficients *R* and *T*. The results obtained are important not only from a theoretical point of view, but also from an applied point of view, since they can be used in practice, for example, to generate quantum entangled photons with specified properties.

## Quantum entanglement and photon statistics

**Figure 1:**
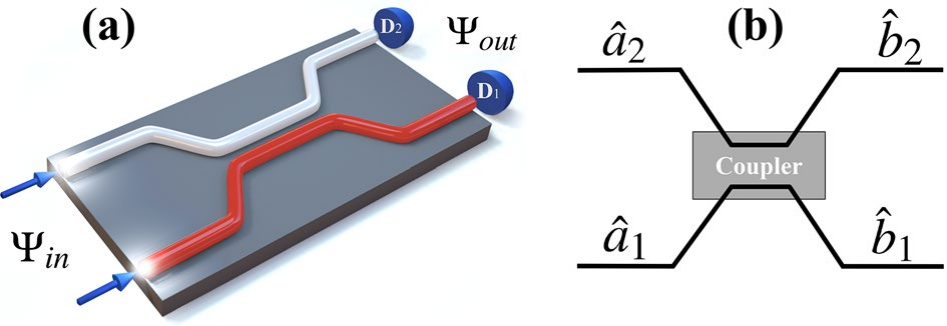
(**a**) 3D representation of the BS in the form of coupled waveguides, where $\Psi _ {in}$ and $\Psi _ {out}$ are the initial and output (recorded by the $D_1$ and $D_2$ detectors) photon wave function, respectively. (**b**) 2D image of such a BS, which is often used in various designs.

It is well known^14,20–22^ that a lossless two-mode BS in quantum optics is described by a unitary matrix $U_{BS}$, which has the form

1 $$\begin{aligned} U_{BS}=\begin{pmatrix} \sqrt{T}&{} e^{i\phi }\sqrt{R}\\ - e^{-i\phi }\sqrt{R}&{} \sqrt{T} \end{pmatrix} ; ~~~ \begin{pmatrix} \hat{b}_1\\ \hat{b}_2 \end{pmatrix}= U_{BS} \begin{pmatrix} \hat{a}_1\\ \hat{a}_2 \end{pmatrix}, \end{aligned}$$

where the annihilation operators 1 and 2 modes at the input to the BS respectively represent $\hat{a}_1$ and $\hat{a}_2$, and after output BS $\hat{b}_1$ and $\hat{b}_2$; *T* and *R* are the coefficients of transmission and reflection; respectively, and $\phi$ is the phase shift. The Eq. (1) is general and applies to any type of linear BS, including a BS in the form of coupled waveguides, see. Figure 1. As was shown in^17,18^, the reflection coefficients *R*, the transmission *T* and the phase shift $\phi$ in the case of the BS in the form of a coupled waveguide will be in the form

2 $$\begin{aligned} R=\frac{\sin ^2\left( \Omega t_{BS}/2 \sqrt{1+\varepsilon ^2} \right) }{(1+\varepsilon ^2)};~T=1-R;~\cos \phi =-\varepsilon \sqrt{\frac{R}{T}} ;~\varepsilon =\frac{\omega _2 -\omega _1}{\Omega }, \end{aligned}$$

where $\Omega$ is a certain frequency characterizing the BS; $t_{BS}$ is the time of interaction of photons in the BS (in the case of monochromatic and identical photons, coincides with^11^, where $R = \sin ^2(C z)$, $\phi = \pi /2$, $C = \Omega /(2 \mathrm{v})$ is the coupling constant between adjacent waveguides, $z= \mathrm{v} t_{BS}$, $\mathrm{v}$ is wave velocity in a waveguide); $\omega _1$ and $\omega _2$ are the photon frequencies in the first and second ports, respectively. It should be added that the greater the coupling in the waveguides, the greater the value of $\Omega$ and vice versa. Thus, we can regulate the coupling in the waveguide by changing $\Omega$.

In general, the matrix $U_ {BS}$ is needed to find the wave function of photons in the output state $\Psi _{out}$ from the initial state $\Psi _{in}$. The wave function $\Psi _ {out}$, taking into account the non-monochromaticity of photons, will be^17,23,24^

3 $$\begin{aligned} \Psi _{out}=\frac{1}{\sqrt{s_1!s_2!}} \int \phi (\omega _1, \omega _2){{\hat{b}_1}^{\dagger }}{}^{s_1} {{\hat{b}_2}^{\dagger }}{}^{s_2}|0\rangle d\omega _1 d \omega _2 \end{aligned}$$

where $s_1$ and $s_2$ are the initial number of photons in modes 1 and 2, respectively, $|0\rangle$ is the vacuum state, and ${\hat{b}_1}$ and ${\hat{b}_2}$ are determined from the BS matrix (1), $\phi (\omega _1,\omega _2)$ is the joint spectral amplitude (JSA) of the two-modes wavefunction ($\int | \phi (\omega _1, \omega _2) |^2 d \omega _1 d \omega _2 = 1$). It should be added that the initial state $\Psi _{in} = \Psi _{out} (t_{BS} = 0) = \int \phi (\omega _1, \omega _2) |s_1, s_2 \rangle d \omega _1 d \omega _2$. It was shown in^17^ that $\Psi _ {out}$ in the case of a BS in the form of coupled waveguides will be

4 $$\begin{aligned} \Psi _{out}=\sum ^{s_1+s_2}_{k=0}\int \phi (\omega _1, \omega _2)c_{k,p}| k, s_1+s_2-k \rangle d\omega _1 d \omega _2 , \end{aligned}$$

where $|k,s_1+s_2-k\rangle =|k\rangle |p\rangle$ is the state of the photons at the output ports of the BS,

5 $$\begin{aligned} c_{k,p}=\sum ^{s_1+s_2}_{n=0}A^{s_1,s_2}_{n,s_1+s_2-n}A^{*{k,p}}_{n,s_1+s_2-n}e^{-2in ~ {\arccos \left( \sqrt{1-R} \sin \phi \right) }} ,~ A^{k,p}_{n,m}=\frac{\mu ^{k+n}\sqrt{m!n!}}{(1+\mu ^2)^{\frac{n+m}{2}}\sqrt{k!p!}}P^{(-(1+m+n), m-k)}_{n}\left( -\frac{2+\mu ^2}{\mu ^2} \right) , \nonumber \\ \mu =\sqrt{1+\frac{1-R}{R}\cos ^2\phi }-\cos \phi \sqrt{\frac{1-R}{R}}, \end{aligned}$$

where $P^{\alpha , \beta }_{\gamma }(x)$ are Jacobi polynomials, $s_1$ and $s_2$ are the number of photons in the first and second input ports, respectively, *k* and *p* are the number of photons in the first and second output ports, respectively. Moreover, the condition $k + p = s_1 + s_2$ is satisfied, i.e. the number of photons in the system does not change^15^, $|k,s_1+s_2-k\rangle =|k\rangle |p\rangle$ is the state of the photons at the output ports of the BS. In this case, the probability $\Lambda _k$ of detecting photons in *k* and $p=s_1 + s_2-k$ states at the first and second ports of the BS, respectively, will be

6 $$\begin{aligned} \Lambda _k= \int |\phi (\omega _1, \omega _2)|^2\lambda _k (R) d\omega _1 d \omega _2 ,~~ \lambda _k (R)=\left| c_{k,s_1+s_2-k}\right| ^2. \end{aligned}$$

Next, we will study the quantum entanglement of such a system. For this, it will be assumed that there is no quantum entanglement of photons at the input ports of the BS. In other words, we will consider the incoming photonic states as Fock, but the photons are not monochromatic. In this case, as is well known, the photon wave function is factorizable, i.e. $\Psi _{in} =\int \phi _1 (\omega _1) |s_1 \rangle d \omega _1 \int \phi _2 (\omega _2) |s_2 \rangle d \omega _2$, where $\phi (\omega _1,\omega _2)=\phi _1(\omega _1) \phi _2(\omega _2)$. It should be added that usually quantum entanglement is considered for monochromatic photons, in this case $\Psi _{in} = |s_1 \rangle |s_2 \rangle$, see e.g.^1,15^. At first glance, it seems that the non-monochromaticity of photons cannot greatly change the quantum entanglement at the BS. As will be shown below, quantum entanglement can vary greatly depending on the degree of non-monochromaticity of the photons. To analyze quantum entanglement, we will use the von Neumann entropy $S_N = - \sum _{k} \Lambda _k \ln \left( \Lambda _k \right)$^1,17,25–27^. The von Neumann entropy $S_N$ is the most commonly used measure in the analysis of quantum entanglement. Next, let’s choose $\phi _i (\omega _i)$ ($i = 1,2$) in the most commonly used form, this is a Gaussian distribution

7 $$\begin{aligned} \phi _i(\omega _i)=\frac{1}{(2\pi )^{1/4}\sqrt{\sigma _i}}e^{-\frac{(\omega _i-\omega _{0i})^2}{4\sigma ^2_i}}, \end{aligned}$$

where $\omega _{0i}$ is the mean frequency and $\sigma _i^2$ is the dispersion. Next, we will use the $\omega _{0i}/\sigma _i \gg 1$ condition, which is applicable to most photon sources. At first glance, it seems that this is enough for photons to be considered monochromatic. This is really so, but only for $\Psi _{in}$, for $\Psi _{out}$ this is no longer the case, since the coefficients *R*, *T* depend on the photon frequencies, which ultimately leads to dependencies of $\Psi _{out}$ from dispersion $\sigma _i$.

**Figure 2:**
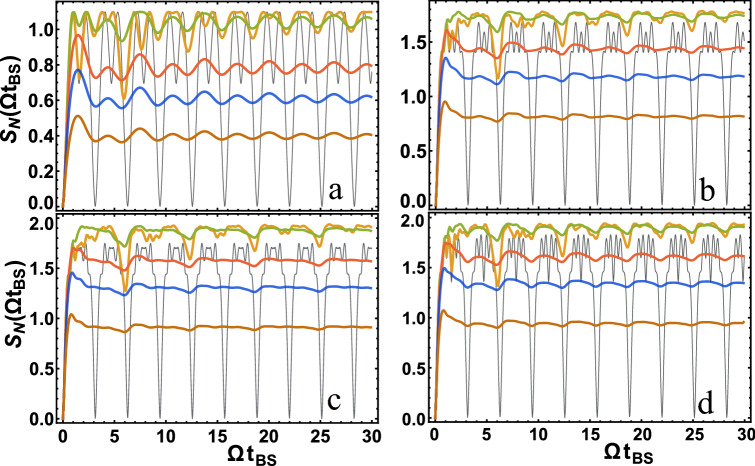
The dependence of the von Neumann entropy $S_N$ on the parameter $\Omega t_ {BS}$ is presented. In (**a**–**d**) the value of $S_N$ is presented for $| 1,1 \rangle , | 2,3 \rangle , | 4,2 \rangle , | 3,3 \rangle$, respectively (where $| s_1, s_2 \rangle$ are input states to 1 and 2 ports of the BS, respectively). All figures show the results for $\sigma / \Omega = 10$ (brown), $\sigma / \Omega = 5$ (blue),$\sigma / \Omega = 3$ (red), $\sigma / \Omega = 1$ (green), $\sigma / \Omega = 1/3$ (orange) from bottom to top, respectively. The thin curve is made at $\sigma / \Omega = 0$ (black).

Figure 2 let us represent the dependence of the von Neumann entropy $S_N$ depending on the dimensionless parameter $\Omega t_{BS}$ for identical photons, i.e. for $\sigma _1 = \sigma _2 = \sigma$ and $\omega _{01} = \omega _{02} = \omega _{0}$.

It should be added that the results obtained for monochromatic photons, i.e. for $\sigma \rightarrow 0$ (more precisely, for $\sigma / \Omega \ll 1$ so that $\sigma t_{BS} \ll 1$) coincide with previously known results, see e.g.^1,15^. Indeed, if we use the result for monochromatic photons^11^, for the reflection coefficient $R = \sin ^2(\Omega t_{BS} / 2)$ and, for example,^1^ for calculating quantum entanglement, then it is easy to get the dependencies presented in Fig. 2 for $\sigma / \Omega = 0$ (thin lines). Thus, our result is more general, applicable to non-monochromatic photons. An interesting and noteworthy result is the large difference between the quantum entanglement of monochromatic and non-monochromatic photons. Moreover, in the case of non-monochromatic photons, when $\sigma / \Omega \sim 1$, the quantum entanglement is larger. The difference is not only in the quantitative value, but also in the qualitative behavior of quantum entanglement. In the case of non-monochromatic photons, when $\sigma / \Omega \gtrsim 1$ and for relatively large $\Omega t_{BS}$, quantum entanglement tends to a constant value. In the case of monochromatic photons, quantum entanglement is a periodic function. This difference is easily explained by comparing the reflection coefficients *R* in the case of monochromatic and non-monochromatic photons, see Eq. (2) and below. In the case of monochromatic photons, this is a periodic function relative to $\Omega t_ {BS}$. In the case of non-monochromatic photons under the sine argument, there is a dependence on the photon frequencies, which ultimately removes the periodicity when integrating over frequencies, see Eq. (6).

**Figure 3:**
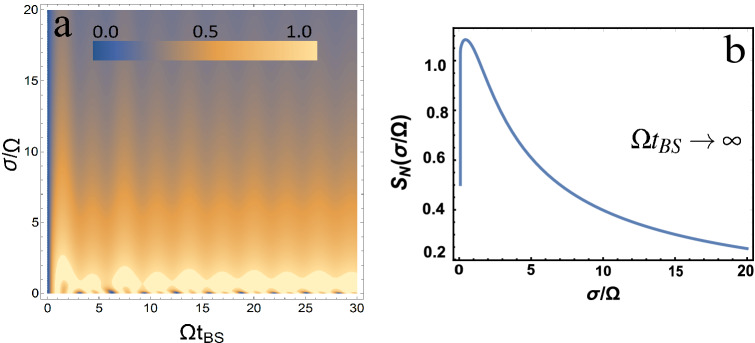
(**a**) A contour plot of the von Neumann entropy $S_N$ versus two parameters $\sigma / \Omega$ and $\Omega t_ {BS}$ for the input state $| 1, 1 \rangle$ is presented. (**b**) von Neumann enetropy $S_N$ is presented as a function of $\sigma / \Omega$ in the limiting case $\Omega t_{BS} \rightarrow \infty$ for the input state $| 1, 1 \rangle$.

Let us consider in more detail the case of input photons in the states $| 1, 1 \rangle$. This case is of particular interest because it implements the Hong-Ou-Mandel (HOM) effect^2,18^. Other cases are close enough in analysis and representations, so it is enough to study the case $| 1,1 \rangle$. To analyze this case, it is convenient to represent the contour plot for the von Neumann entropy depending on two parameters $\sigma / \Omega$ and $\Omega t_{BS}$, see Fig. 3(a). Since, for large $\Omega t_{BS}$ (of course, the condition $\sigma t_ {BS} \gg 1$ must also be satisfied) quantum entanglement tends to a constant value, then Fig. 3(b) represents this constant value, depending on the $\sigma / \Omega$ parameter. Moreover, you can find the analytical dependence represented by the dependence Fig. 3(b) as

8 $$\begin{aligned} S_N=\ln \frac{2(1-\mathrm{J})^{\mathrm{J}-1}}{(2\mathrm{J})^{\mathrm{J}}} ,~~ \mathrm{J}=1+\frac{3}{8}\left( \frac{\Omega }{\sigma }\right) ^2-\frac{\sqrt{\pi }}{16} \left( \frac{\Omega }{\sigma }\right) ^3 \left\{ 3+10 \left( \frac{\sigma }{\Omega }\right) ^2 \right\} \mathrm{erf}\left( \frac{\Omega }{2\sigma }\right) {e}^{\left( \frac{\Omega }{2\sigma }\right) ^2} , \end{aligned}$$

where $\texttt {erf}$ is an error function. Should be added that, to get the Eq. (8) sine and cosine terms (rapidly oscillating terms at $\Omega t_{BS} \rightarrow \infty$) must be ignored when integrating over frequencies.

It can be seen from the graphs obtained that the quantum entanglement has a maximum, which will be at $\sigma / \Omega = 0.44467$. It is also seen that the quantum entanglement is large at $\sigma / \Omega \sim 1$, and as $\sigma / \Omega$ increases, it tends to zero.

**Figure 4:**
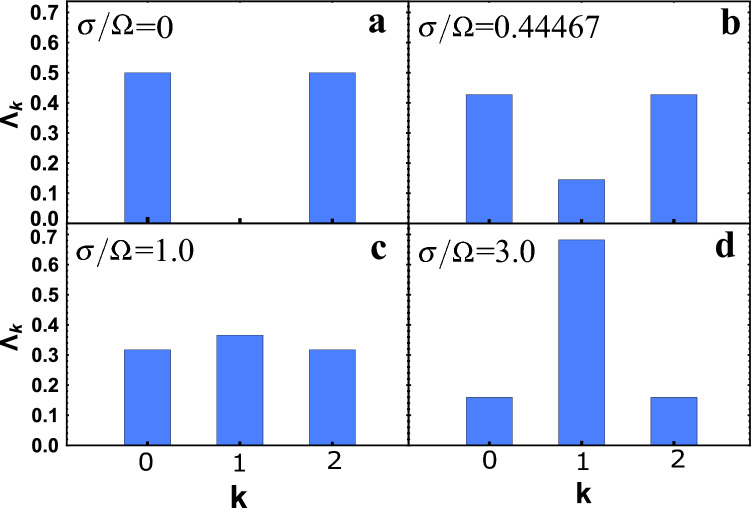
A histogram of the dependence of the probability $\Lambda _k$ of detecting *k* and $p = s_1 + s_2-k$ (we consider the case $| 1,1 \rangle$, where $s_1 = s_2 = 1$) photons at the output of the first and second ports, respectively, for different values of $\sigma / \Omega$.

Next, consider the statistics of photons at the output ports of the BS. This requires a probability analysis $\Lambda _k$ see Eq. (6). If we were considering monochromatic photons, but the coefficient *R* would be constant, then following Eq. (6) $\Lambda _k = \lambda _k$. This case was discussed in detail earlier, see e.g.^14^. You can see that our results, in this case, match^14^. Thus, our consideration is general and, in a particular case, coincides with the previously well-known results. Consider a BS with specified characteristics. Let us choose its length $z = v t_{BS}$ and frequency $\Omega = 2 C v$ (see above, after Eq. (2)) so that the reflection coefficient for monochromatic and identical photons is $R = \sin ^2 (\Omega t_{BS}/2) = 1/2$. As a result, we choose $\Omega t_{BS} = 5 \pi / 2$. This choice is determined by how the statistics of photons change depending on their non-monochromaticity, which can be seen on the example of a particular BS. In addition, the choice of the reflection coefficient $R = 1/2$ is found in many applications of quantum optics^2,28,29^. Thus, further we will consider identical ($\sigma _1 = \sigma _2 = \sigma$ and $\omega _{01} = \omega _{02} = \omega _{0}$), but not monochromatic photons.The calculation results are presented in Fig. 4. From Fig. 4(a) you can see that for the states $| 1,1 \rangle$ at $\sigma / \Omega = 0$ the HOM effect^2^ is realized. This means that only pairs of photons are recorded on the first or second detectors (in the figure, this is for $k = 0$ and $k = 2$) with a probability of 1/2. By increasing $\sigma / \Omega$ the HOM effect disappears and the photon statistics changes dramatically. It is quite interesting to see the statistics of photons at maximum quantum entanglement, see Fig. 4(b). This statistic differs from the statistic of the HOM effect Fig. 4(a) and from statistics in the case of monochromatic photons at maximum quantum entanglement, i.e. with constant reflection coefficient *R*, see^15^. For large $\sigma / \Omega \gg 1$, the probability $\Lambda _k$ will tend to unity for $k = 1$. This means that photons will never arrive in pairs at the detectors. A similar analysis is quite simple to carry out for any input states $| s_1, s_2 \rangle$. A common thing for all cases will be this coincidence with the statistics for monochromatic photons at $\sigma / \Omega = 0$ presented in the work^14^, see also^15^. Also, the general behavior of the probability for any states $| s_1, s_2 \rangle$ this will be the tendency of the photon statistics at $\sigma / \Omega \gg 1$ to the statistics of incoming photon states. This is easily explained, since at $\Omega \rightarrow 0$ (the same as $\sigma / \Omega \gg 1$), the coupling between the waveguides in the BS weakens, which means that photons propagate along their waveguides.

**Figure 5:**
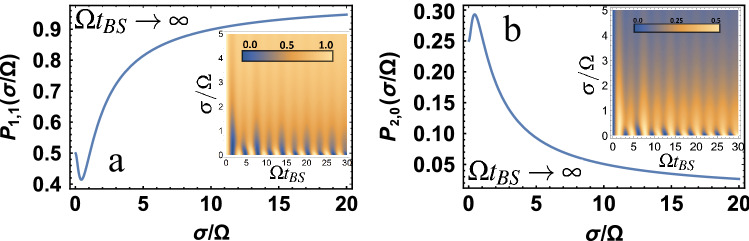
(**a**) Shows the probability $P_{1,1}$, (**b**) shows the probability $P_{2,0} = P_ {0,2}$ for $\Omega t_{BS} \rightarrow \infty$ depending on the $\sigma / \Omega$ parameter. Also in the insets (**a**),(**b**) are contour plots for the probability $P_{1,1}$ and $P_ {2,0} = P_ {0,2}$ are presented, respectively, depending on two parameters of the studied system $\sigma / \Omega$ and $\Omega t_{BS}$.

Also consider the limiting case described above for quantum entanglement, this is the case for large $\Omega t_ {BS}$ (more precisely, the condition $\sigma t_{BS} \gg 1$ must also be satisfied) for the input state $| 1,1 \rangle$. In this case, it is easy to obtain the probability of detecting photons on both detectors $P_{1,1} = \mathrm{J}$, where $\mathrm{J}$ is represented in Eq. (8). The probability of detecting pairs of photons on 1 or 2 detectors will then be $P_ {2,0} = P_ {0,2} = 1/2 (1-P_ {1,1})$. The results are presented in Fig. 5. It is interesting enough to note that the minimum of the function $min \{P_{1,1} \}$ or the maximum of $max \{ P_{2,0} = P_{0,2} \}$ for $\sigma / \Omega = 0.44029$ practically coincides with the maximum for quantum entanglement at $\sigma / \Omega = 0.44467$ (see Fig. 3(b)). This means that the maximum of quantum entanglement is realized when photons at the first and second detectors can be registered with a minimum probability. Or, which is the same, when pairs of photons can be recorded on detectors with maximum probability. Also in the insets Fig. 5(a),(b) the probabilities $P_{1,1}$ and $P_ {2,0} = P_ {0,2}$ are presented, respectively, depending on two parameters of the studied system $\sigma / \Omega$ and $\Omega t_{BS}$. It can be seen that taking into account the non-monochromaticity of photons significantly changes the probabilities, in comparison with monochromatic ones.

It should be added that such an analysis, for quantum entanglement and photon statistics, is fairly easy to carry out for any photons input states $| s_1, s_2 \rangle$, as well as various BS parameters $\Omega , t_{BS}$ and the values of non-monochromaticity of photons $\sigma _1, \sigma _2$.

## Discussion and Conclusion

Thus, we have studied quantum entanglement and photon statistics on a BS based on coupled waveguides. Coupled waveguides are frequency-dependent BS, i.e. reflection coefficients *R* and transmission *T* depend on the frequencies of incoming photons to ports 1 and 2 of the BS. Since our theory takes into account the frequency dependence of the coefficients *R* and *T*, it means that we take into account the non-monochromaticity of input photons. One of the main conclusions is a significant difference between quantum entanglement and photon statistics compared to a BS, where *R* and *T* do not depend on frequencies. Given that the quantum entanglement and statistics of photons of a frequency-dependent BS have not been studied previously, this conclusion is very important. If we choose *R* and *T* as constant values, then our theory in the limiting case is $\sigma / \Omega \rightarrow 0$ (in general, $\varepsilon \rightarrow 0$ in Eq. 2 ) coincides with the previously well-known theories, for quantum entanglement e.g.^1,15^ and photon statistics e.g.^14^. It should be added that Fock’s input states are considered here, since they are not quantum entangled. In general, this theory can be easily generalized to arbitrary input states, including quantum-entangled states, see eg^30,31^.

The frequency-dependent BS discussed here can be a good source of quantum entangled photons. Moreover, quantum entanglement is easy to regulate by changing the $\Omega$ parameter (see e.g. Fig. 3(a)). The $\Omega$ parameter is quite easy to change, following the work^17,18^. To do this, you just need to weaken or increase the connection in the waveguides. This can be done, for example, by separating or bringing the waveguides closer together. A big advantage in using a frequency-dependent light splitter as a source of quantum entanglement of photons is practically the maximum possible quantum entanglement for $\sigma / \Omega \sim 1$ and $\Omega t_{BS}> 1$. As is well known e.g.^1,32^ that the maximum quantum entanglement for the von Neumann entropy $S_N = \ln (1 + N)$ when $N + 1$ is a dimensional bipartite system. In our case, $N = s_1 + s_2$^1^. It should also be added that in this work (similar to eg^1^), quantum entanglement of photons is understood as bipartite entanglement of modes, see eg^33^. In contrast to the case that is realized on a non-frequency-dependent BS (where *R* and *T* are constants), in our case, for $\sigma / \Omega \sim 1$ and $\Omega t_{BS}> 1$, quantum entanglement is close to its maximum value. For constant *R* and *T*, quantum entanglement is a periodic function with respect to $\Omega t_{BS}$, and for large $\Omega t_{BS} \gg 1$, it is a rapidly oscillating dependence.

All this suggests that a frequency-dependent beam splitter based on coupled waveguides can be used as a source of large quantum entanglement of photons. The results obtained can have interesting practical applications in quantum optics, in particular, in quantum metrology and quantum information.
